# Successful induction treatment of bullous pemphigoid using reslizumab: a case report

**DOI:** 10.1186/s13223-021-00619-1

**Published:** 2021-11-16

**Authors:** Hyo-In Rhyou, Song-Hee Han, Young-Hee Nam

**Affiliations:** 1 Department of Internal Medicine, Samsung Changwon Hospital, Sungkyunkwan University School of Medicine, Changwon, Korea; 2 Department of Pathology, Dong-A University College of Medicine, Dong-A University Hospital, Busan, Korea; 3 Department of Internal Medicine, College of Medicine, Dong-A University Hospital, 26 Daesingongwon-ro, Seo-gu, Busan, Korea

**Keywords:** Bullous pemphigoid, Reslizumab, Eosinophil, Corticosteroid

## Abstract

**Background:**

Bullous pemphigoid (BP) is a potentially life-threatening autoimmune blistering disease which is characterized by autoantibodies against hemidesmosomal proteins of the skin and mucous membranes. In recent years, the role of eosinophil and immunoglobulin E autoantibodies have been further elucidated in BP, and have been considered potential therapeutic targets.

**Case presentations:**

A 67-year-old male presented with erythematous bullous eruption. The skin eruption was located on whole body, and suggested BP. Peripheral blood eosinophil count and total immunoglobulin E markedly elevated in initial laboratory findings. Topical and systemic steroid (methylprednisolone 2 mg/kg/day) treatment was started, and his skin symptoms worsened repeatedly, whenever systemic steroid were reduced. On admission day 29, reslizumab (anti-interleukin-5) 3.5 mg/kg was administered intravenously to the patients. The bullous skin lesion began to improve rapidly, and methylprednisolone (8 mg/day) was reduced without any worsening of symptoms during two doses of reslizumab.

**Conclusions:**

We report a case of successful treatment response to reslizumab administration in a patient with BP. Further studies are needed to confirm the role of anti-interleukin-5 as a treatment for BP in the future.

## Introduction

Bullous pemphigoid (BP) is a potentially life-threatening autoimmune blistering disease characterized by the development of autoantibodies against the BP180 and BP230 hemidesmosomal proteins in the skin and mucous membranes [1, 2]. Topical and/or systemic corticosteroids are the mainstay of therapy for BP. Corticosteroid-sparing agents should be considered in patients in whom BP is not effectively controlled with the exclusive administration of corticosteroids or in patients who develop corticosteroid-induced adverse effects [3].

BP typically presents with peripheral blood eosinophilia and tissue eosinophils, and the role of eosinophils in BP has been investigated in detail [4]. Recent studies have reported that eosinophils induce dermal-epidermal separation in the presence of BP autoantibodies and these cells are therefore being viewed as potential therapeutic targets in patients with BP [4, 5].

## Case

**Fig. 1:**
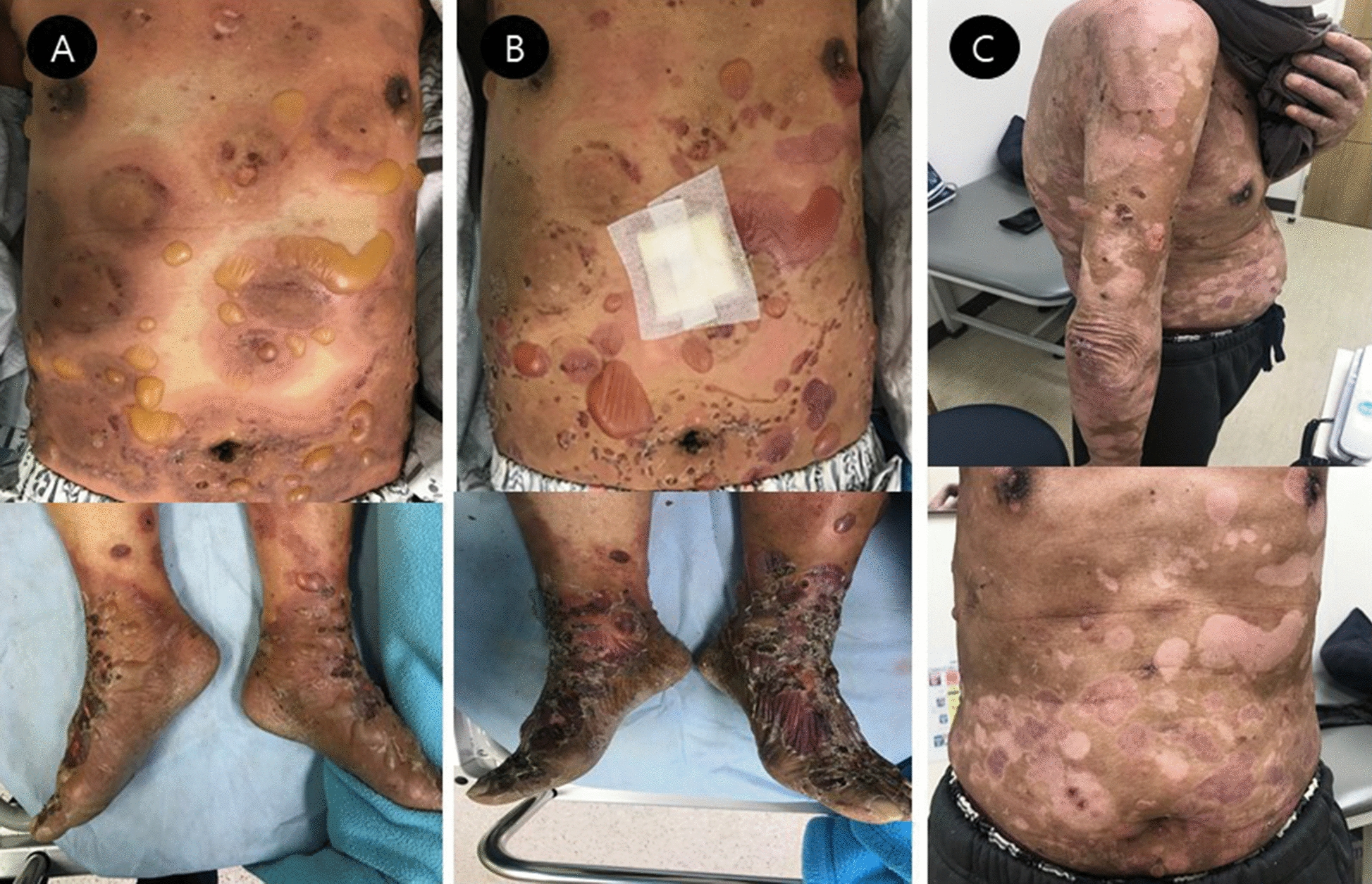
Image showing a skin lesion suggestive of bullous pemphigoid (**A** on admission day). No improvement in the skin lesion is observed during steroid treatment (**B** on admission day 14). Significant improvement in the lesion is observed after the administration of reslizumab [**C** on the day of discharge (day 14 after admission)]

**Fig. 2:**
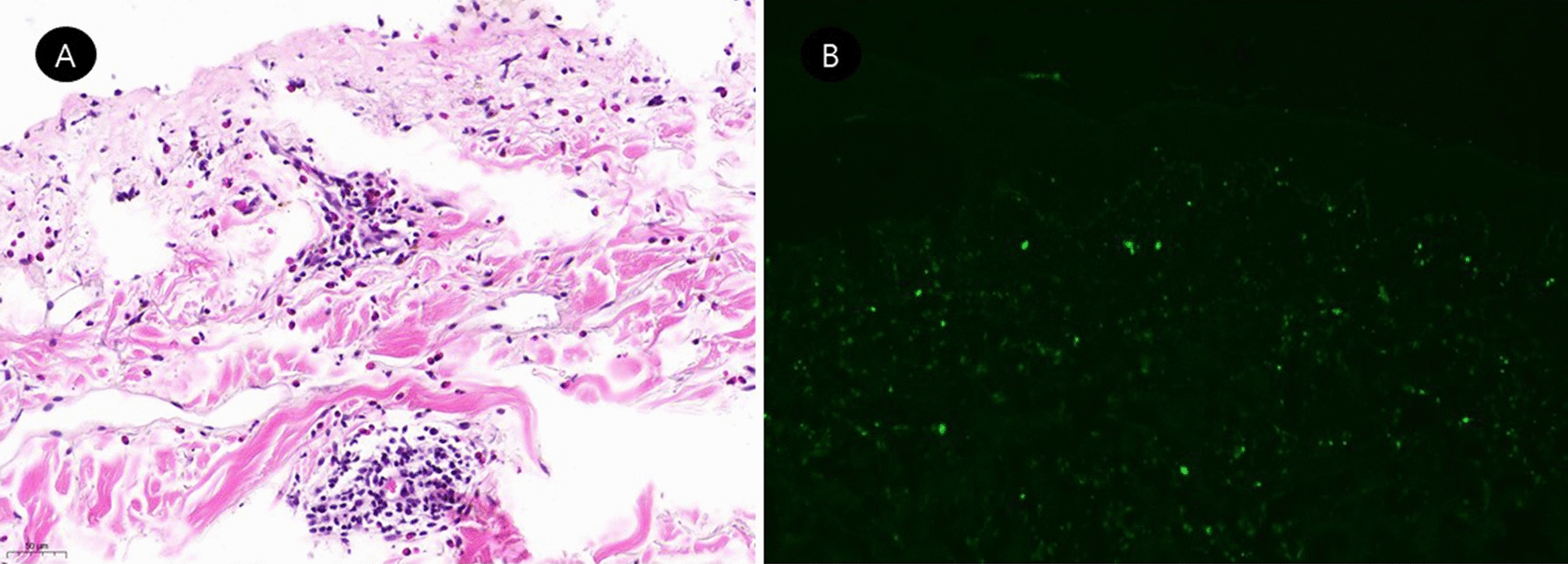
Histopathological examination of the patient. subepidermal bulla with perivascular inflammatory cell infiltration with some eosinophils were shown in histologic examination of a skin biopsy of a bullous lesion on the abdomen (**A** H&E:  × 364). Direct immunofluorescence showing granular deposits of complement C3 along the basement membrane (**B**  × 100)

A 67-year-old man with no significant medical history presented with a 2-month history of an erythematous bullous eruption throughout the body, which suggested a diagnosis of BP (Fig. 1A). A punch biopsy performed on admission day 2 revealed a subepidermal bulla with perivascular inflammatory cell infiltration with some eosinophils (Fig. 2A). Direct immunofluorescence microscopy of the skin revealed granular deposition of complement C3 along the basement membrane (Fig. 2B). Laboratory test findings showed an elevated whole blood count of 22,010 cells/μL and a peripheral blood eosinophil count of 5,062 cells/μL. The serum total immunoglobulin (Ig) E level was significantly elevated (1,580 KU/L). C-reactive protein (CRP) was normal (0.3 mg/dL; normal range  < 0.5 mg/dL). Topical and systemic steroid (methylprednisolone 2 mg/kg/day) administration was initiated on admission day 2. However, his skin symptoms worsened, following reduction in the systemic steroid dose (Fig. 1B). He received a weekly dose of methotrexate (5 mg, orally) on admission day 10. However, he complained of febrile sensation and general weakness, and CRP was elevated (3.37 mg/dL). His skin symptoms were not improved at all, and methotrexate was discontinued after only one dose. He received a subcutaneous injection of omalizumab (300 mg) on admission day 15; however, we did not observe a satisfactory systemic steroid-sparing effect. Reslizumab, an anti-interleukinr-5 (IL-5) antibody, 3.5 mg/kg was administered intravenously on admission day 29, which minimized itching, and his blood eosinophil count decreased from 1,300 cells/μL to 0 cells/μL the following day. The bullous skin lesion improved rapidly, and he was discharged after a prednisolone taper to 40 mg/day (Fig. 1C). The patient received the same dosage of reslizumab after 4 weeks, and methylprednisolone (8 mg/day) was reduced without any worsening of symptoms. He stopped two doses of reslizumab and cyclosporine was started. However, worsening of skin lesions necessitated an increase in the steroid dose.

## Discussion

BP is the most common autoimmune blistering skin disease that predominantly affects elderly individuals [1, 2]. Blister formation in BP is attributed to the formation of IgG autoantibodies against the hemidesmosomal proteins BP180 and BP230 [1, 2]. However, many of the clinical manifestations and pathways associated with BP cannot be sufficiently explained exclusively by the IgG autoantibody-mediated immunologic response against the BP180 and BP230 proteins [4, 6]. BP is characterized by peripheral eosinophilia and eosinophilic tissue infiltration [4]. The exact role of eosinophils in the pathogenesis of BP remains unknown, although several recent studies implicate eosinophils as a contributor to BP [4, 5, 7]. Eosinophils bind the anti-BP180 IgG via high-affinity IgE receptor expression and lead to dermal–epidermal junction separation [4, 8]. Eosinophils secrete matrix metalloproteinase-9, which is known to cause cleavage of BP180 [9]. Additionally, eosinophils secrete IL-31, which is a well-known pruritogen in BP [10] and may serve as a functional antigen-presenting cell [11, 12]. Research has highlighted the role of eosinophils as a major etiopathogenetic contributor to BP; therefore, they are being considered potential therapeutic targets [7]. Anti-IL-5 antibodies block IL-5 and reduce eosinophil levels of blood, bone marrow and tissue, and some studies have shown the efficacy and safety of these agents in patients with allergic diseases and hypereosinophilic syndrome [13–15]. However, the effects of anti-IL-5 antibodies in patients with BP remain unclear.

Topical and systemic corticosteroid treatment is the mainstay for management of BP. Adjuvant immunosuppressive drugs such as methotrexate, azathioprine and cyclophosphamide are indicated for patients who need high doses corticosteroids to control disease or who have adverse effects on corticosteroid treatment [1, 2]. In recent years, biologics such as rituximab and omalizumab may be effective to BP with better safety profile than classical immunosuppressive drugs [1, 2, 16, 17]. Rituximab is a monoclonal antibody against the protein CD-20 antigen on B lymphocyte. Omalizumab is a monoclonal antibody binding to free human IgE, and downregulates cell surface IgE receptors of mast cell, basophil, and dendritic cell as well as eosinophil. The mechanisms of action of drugs are different. Therefore, it is considered that an appropriate patient selection may be important [16, 17]. The patient in this case had BP that was refractory to high-dose systemic steroid therapy and methotrexate, and there were the potential risks of infections for using classical immunosuppressive adjuvant drugs. He presented with high levels of serum total IgE and blood eosinophil count, known as good predictors of omalizumab therapy in allergic asthma, [18] but there were no clinical improvements. It was difficult to wait completely confirm the treatment response to these drugs because of the deterioration of the patient’s general condition and extensive skin lesions. However, blood eosinophilia and the patient’s symptoms improved rapidly from the day after reslizumab administration, and re-exacerbation of the skin lesion was noted after discontinuation of reslizumab. Therefore, we assumed that the clinical improvement of the patient might be the effect of reslizumab, and this is thought to be because eosinophil may be an end stage effector cell of BP.

A clinical study has reported the effects of anti-IL-5 therapy in patients with BP [19]. A randomized, placebo-controlled, double-blind phase 2 pilot study in Switzerland reported that mepolizumab was ineffective against BP with regard to the clinical outcomes and the percentages of patients who showed no BP relapse. The difference between the outcomes of the aforementioned study and those observed in our study was attributable to the fact that the endpoint in that study was the cumulative rate of relapse-free patients after mepolizumab treatment and not the steroid-sparing effect.

## Conclusions

This is a case of successful treatment of BP using anti-IL-5 antibody (reslizumab). Future research is warranted to confirm the role of biologics such as anti-IL5 as a therapeutic option for BP, particularly with regard to the steroid-sparing effect.

## Data Availability

All data generated or analyzed during this study are included in this published article.

## References

[1] Miyamoto D, Santi CG, Aoki V, Maruta CW (2019). Bullous pemphigoid. An Bras Dermatol.

[2] Moro F, Fania L, Sinagra JLM, Salemme A, Zenzo GD (2020). Bullous pemphigoid: trigger and predisposing factors. Biomolecules.

[3] de Vega IF, Iranzo-Fernández P, Mascaró-Galy JM (2014). Bullous pemphigoid: clinical practice guidelines. Actas Dermosifiliogr.

[4] Amber KT, Valdebran M, Kridin K, Grando SA (2018). The role of eosinophils in bullous pemphigoid: a developing model of eosinophil pathogenicity in mucocutaneous disease. Front Med.

[5] Lin L, Hwang BJ, Culton DA, Li N, Burette S, Koller BH (2018). Eosinophils mediate tissue injury in autoimmune skin disease bullous pemphigoid. J Invest Dermatol.

[6] Genovese G, Zenzo GD, Cozzani E, Berti E, Cugno M, Marzano AV (2019). New insights into the pathogenesis of bullous pemphigoid: 2019 update. Front Immunol.

[7] Simon D, Borradori L, Simon HU (2017). Eosinophils as putative therapeutic targets in bullous pemphigoid. Exp Dermatol.

[8] Messingham KN, Wang JW, Holahan HM, Srikantha R, Aust SC, Fairley JA (2016). Eosinophil localization to the basement membrane zone is autoantibody- and complement-dependent in a human cryosection model of bullous pemphigoid. Exp Dermatol.

[9] Stahle-Backdahl M, Inoue M, Guidice GJ, Parks WC (1994). 92-kD gelatinase is produced by eosinophils at the site of blister formation in bullous pemphigoid and cleaves the extracellular domain of recombinant 180-kD bullous pemphigoid autoantigen. J Clin Invest.

[10] Kunsleben N, Rüdrich U, Gehring M, Novak N, Kapp A, Raap U (2015). IL-31 induces chemotaxis, calcium mobilization, release of reactive oxygen species, and CCL26 in eosinophils, which are capable to release IL-31. J Invest Dermatol.

[11] Wang HB, Ghiran I, Matthaei K, Weller PF (2007). Airway eosinophils: allergic inflammation recruited professional antigen-presenting cells. J Immunol.

[12] Lin A, Lore K (2017). Granulocytes: new members of the antigen-presenting cell family. Front Immunol.

[13] Nagase H, Ueki S, Fujieda S (2020). The roles of IL-5 and anti-IL-5 treatment in eosinophilic diseases: asthma, eosinophilic granulomatosis with polyangiitis, and eosinophilic chronic rhinosinusitis. Allergol Int.

[14] Mukherjee M, Sehmi R, Nair P (2014). Anti-IL5 therapy for asthma and beyond. World Allergy Organ J.

[15] Hassani M, Koenderman L (2018). Immunological and hematological effects of IL-5(Rα)-targeted therapy: an overview. Allergy.

[16] Kremer N, Snast I, Cohen ES, Hodak E, Mimouni D, Lapidoth M (2019). Rituximab and omalizumab for the treatment of bullous phemphigoid: a systemic review of the literature. Am J Clin Dermatol.

[17] Bernard P, Antonicelli F (2017). Bullous pemphigoid: a review of its diagnosis, associations and treatment. Am J Clin Dermatol.

[18] Agache L, Akdis CA, Akdis M, Canonica GW, Casale T, Chivato T (2021). EAACI biological guidelines-recommendations for severe asthma. Allergy.

[19] Simon D, Yousefi S, Cazzaniga S, Bürgler C, Radonjic S, Houriet C (2020). Mepolizumab failed to affect bullous pemopigoid: a randomized, placebo-controlled, double-blind phase 2 pilot study. Allergy.

